# Implementation of an Intervention Program Based on Virtual Walking and Therapeutic Exercise in Cuba: A Feasibility Study

**DOI:** 10.3390/healthcare14030352

**Published:** 2026-01-30

**Authors:** Noemí Moreno-Segura, Sara Mollà-Casanova, Elena Muñoz-Gómez, Héctor González-Pons, Marta Inglés

**Affiliations:** Unitat de Recerca en Biomecànica Clínica—UBIC Research Group, Department of Physiotherapy, Faculty of Physiotherapy, University of Valencia, 46010 Valencia, Spain; noemi.moreno@uv.es (N.M.-S.); elena.munoz-gomez@uv.es (E.M.-G.); hector.gonzalez@uv.es (H.G.-P.); marta.ingles@uv.es (M.I.)

**Keywords:** cooperation, Virtual Walking, mirror neuron therapies, therapeutic exercise, rehabilitation

## Abstract

**Background/Objectives:** This article presents the feasibility and preliminary outcomes of an international cooperation project between the University of Valencia (Spain) and the University and health authorities of Pinar del Río (Cuba), designed to implement and evaluate an innovative rehabilitation protocol. Aligned with the United Nations Sustainable Development Goals (SDGs 3, 4, and 10), the initiative aims to implement a low-cost, evidence-based rehabilitation program combining mirror-neuron stimulation via Virtual Walking and therapeutic exercise. **Methods:** The program included multidisciplinary meetings and both digital and on-site training for healthcare professionals, caregivers, and educators, aimed at strengthening local capacities in evidence-based practice. The transferred protocol consisted of Virtual Walking (10 min) and therapeutic exercise (30 min), implemented three times per week, for eight weeks. Outcomes assessed included gait speed and endurance (10-Minute Walking Test, 6-Minute Walking Test), lower limb function (Timed Up and Go Test), frailty status (Fried criteria), pain (Visual Analog Scale), and satisfaction with the training program. Pre-post comparisons were conducted using the Wilcoxon signed-rank test for continuous data. **Results:** The program was successfully implemented in two polyclinics with high levels of participant satisfaction. Eleven patients completed the program, showing significant improvements in gait endurance (*p* < 0.05), while lower limb function and pain did not change significantly. Noteworthily, severe infrastructural and connectivity limitations were found. Overall, results demonstrate the feasibility, adaptability, and acceptability of the proposed protocol, which integrates technological innovation, clinical training, and community engagement to promote health quality and equity. **Conclusions:** This project provides a replicable framework for rehabilitation initiatives in low-resource settings and demonstrates the potential to achieve meaningful clinical results.

## 1. Introduction

Ensuring equitable access to quality health care remains a paramount challenge for health systems worldwide [1]. In recent years, international cooperation initiatives have played a critical role in strengthening healthcare delivery in resource-limited settings, such as Cuba [2]. These initiatives are aligned with the United Nations Sustainable Development Goals (SDGs), particularly Goal 3 (Good Health and Well-being), Goal 4 (Quality Education), and Goal 10 (Reduced Inequalities), which emphasize the imperative of guaranteeing universal health coverage, equitable access to knowledge, and inclusive strategies for vulnerable populations [3].

Cuba faces persistent and profound socioeconomic challenges, characterized by extreme disparities in wealth distribution and limited access to information across its population [4]. According to a recent independent assessment, the country is currently experiencing its most severe economic crisis since independence, with an estimated 88–89% of the population living below the extreme poverty threshold as of 2023–2024 [1,5]. Social rights studies highlight acute shortages of essential goods, widespread food insecurity, and escalating inflation, further deteriorating living conditions for millions of Cubans [1]. In the healthcare sector, despite a strong emphasis on primary care and regionally organized services, the system remains constrained by limited resources, infrastructural deficits, and accessibility barriers, particularly in rural municipalities. Rehabilitation services are further compromised by material and professional training limitations, restricting access to evidence-based clinical protocols and opportunities for older adults and persons with disabilities [1].

On the other hand, Cuba has one of the oldest demographic profiles in Latin America, with 15.73% of its population aged 65 or older and a mean life expectancy of 78 years [6]. Approximately 7% of Cubans live with disabilities, with physical–motor impairments representing the second most prevalent category (8.25 per 1000 inhabitants) [1]. The demographic transition towards an aging population, compounded by the emigration of younger cohorts seeking better opportunities abroad, has intensified the burden of the Cuban health system [1]. In this context, Pinar del Rio serves as a representative province, reflecting national trends in poverty, suboptimal healthcare provision, and demographic ageing.

Aging populations and individuals with disabilities—particularly those with chronic neurological conditions—pose substantial challenges for healthcare delivery [7]. Sensory and motor deficits frequently lead to functional decline, with downstream consequences including dependency, reduced ability to perform activities of daily living, social isolation, sedentary behavior, cognitive deterioration, and increased healthcare expenditure [8,9]. These observations underscore the urgent need for low-cost, evidence-based therapeutic interventions that enhance functional capacity while alleviating the burden on families, communities, and healthcare systems. While exercise remains the first-line intervention, these individuals often experience early-onset and pronounced fatigue, which may limit adherence to rehabilitation programs and increase dropout risk. This necessitates careful tailoring of the exercise prescription, including volume and intensity, to the low baseline physical fitness commonly observed in this population. In this context, emerging evidence indicates that non-fatiguing interventions, such as bodily illusion-based therapies, can improve pain and functional outcomes in populations with diverse neurological impairments by activating the mirror neuron system [10]. Mechanistically, this system comprises a network of neurons in motor and premotor cortical regions that are active not only during movement execution but also during the observation of others’ goal-directed actions [11,12]. Repeated observation of purposeful movements visually integrated with the individual’s own body representation may facilitate motor resonance, drive cortical reorganization, and support motor learning, even in the presence of impaired voluntary movement [11,12].

Neuroplasticity can be potentiated by combining exercise, the cornerstone of rehabilitation, with mirror-neuron-targeted interventions. The most used mirror-neuron activation therapies are motor imagery, mirror therapy, and action observation [13]. Bodily illusion-based interventions have also demonstrated efficacy across diverse clinical populations. For instance, “Virtual Walking” is a specific bodily illusion-based intervention in which participants visually perceive gait-related lower-limb movements integrated with their reflected upper body, creating the illusion of a fully functional body. Unlike traditional Mirror Therapy, which relies on unilateral limb reflection and requires an intact contralateral side, Virtual Walking can be applied to conditions involving bilateral motor impairments [14]. This innovative intervention was initially tested in people with complete spinal cord injury, and due to the promising results, its effectiveness was subsequently tested in other populations [15].

Virtual Walking combines continuous visual exposure to walking movements with active postural engagement and the illusion of body ownership, while maintaining minimal physical demand [16]. This approach activates motor-related neural circuits without requiring repetitive real-world gait practice, which may be poorly tolerated by frail and pre-frail neurological patients [17]. Despite this potential, clinical evidence from real-world settings remains limited. A scalable rehabilitation protocol, adapted to local contexts and implemented in regional clinics, could significantly improve access and the effectiveness of Cuban healthcare, particularly for populations isolated by geographic or socioeconomic barriers. Integrating such interventions within the broader SDG framework underscores their relevance for sustainable health and social equity [3].

To address these multifaceted challenges, the current cooperation project between the University of Valencia (UV) and the University and health authorities of Pinar del Rio aimed to achieve the following:

1. Implement a mirror-neuron stimulation protocol combining Virtual Walking and therapeutic exercise to enhance functional outcomes in older adults and individuals with neurological conditions.
2. Provide comprehensive training for healthcare professionals, educators, and caregivers in evidence-based functional recovery strategies.
3. Foster interdisciplinary dialog and collaborative learning among medical and academic communities in both countries.
4. Establish robust local partnerships to ensure sustainable implementation of low-cost, high-impact rehabilitation services.

This report describes the clinical intervention protocol, summarizes preliminary outcomes, and discusses implications for future rehabilitation policy and healthcare in Cuba.

## 2. Materials and Methods

### 2.1. Study Design and Setting

This collaborative project was conducted between the UV and the University and health authorities of Pinar del Río, Cuba, from September 2024 to December 2025. The study was approved by the Ethics Committee of the University of Valencia (1304149) and performed in accordance with the latest revision of the Declaration of Helsinki.

The initiative encompassed multidisciplinary meetings, virtual and face-to-face training sessions, installation of Virtual Walking and therapeutic exercise resources, and implementation of the intervention protocol. Activities were developed in partnership with the Municipal Health Sector and representatives from local rehabilitation centers in Pinar del Río. Project activities were organized into five operational periods: (1) Project definition, planning of the timelines, and recruitment of frail and prefrail older adults and individuals with neurological disorders; (2) Material acquisition and recording of online training content; (3) On-site intervention and practical workshops conducted by two UV researchers who traveled to Cuba; (4) Assessment of participant satisfaction with the training and evaluation of the intervention effects on the target populations; (5) Dissemination of results.

### 2.2. Collaborative Meetings

A total of eight preparatory and coordination meetings were held with local Cuban clinical and academic partners prior to protocol implementation, and three additional meetings were conducted after the on-site stay. Topics addressed included project objectives, action plans, identification of local needs, available resources, logistical arrangements for training and intervention deployment, and other practical issues related to the stay in the host country and planned activities. A detailed summary of topics addressed in each meeting is provided in Table 1. The local team consisted of five professionals from provincial and municipal health authorities, as well as academic leaders responsible for teaching, research, and international relations. The UV team comprised six professors from the Physiotherapy Department, including two coordinators and four team members.

### 2.3. Equipment Provided

Essential materials were delivered, including projection screens, two stands, two projectors, two laptops, mirrors, portable examination tables, BOSU^®^ trainers, steps, cones, wrist and ankle weights (1 kg and 2 kg), dynamometers, pulse oximeters, and additional equipment for functional testing and therapeutic exercise implementation.

### 2.4. Training Program

A collaborative, open-access virtual training platform was designed and hosted by UV to facilitate education in functional recovery strategies. Virtual training was delivered asynchronously to avoid difficulties related to time zone differences between countries. The content was addressed to healthcare professionals and caregivers, who were granted full access through a permanent link. Training materials consisted of explanatory videos that covered the following areas: therapeutic exercise (general principles, applications in older adults, and interventions for individuals with musculoskeletal, cardiorespiratory, nervous system and pelvic floor disorders); mirror neuron activation therapies; neurodynamic; and ergonomics and special care for individuals with functional limitations, with a particular focus on caregivers. Content customisation was guided by participant feedback and evolving educational needs. A total of twelve narrated presentations covering nine distinct educational topics were recorded. A Moodle-based platform was developed to allow trainees to access materials and manage learning at their own pace. Asynchronous communication was supported through discussion forums and direct messaging. Furthermore, all training content was stored on external USB drives and distributed during the on-site visits of two UV researchers to mitigate connectivity limitations. Most households and healthcare clinics in rural Pinar del Río lack reliable Wi-Fi access, significantly limiting the use of online resources. To address this constraint, joint video sessions and facilitated peer-learning workshops were organized using the projectors and screens provided, ensuring collective access to educational materials.

**Figure 1:**
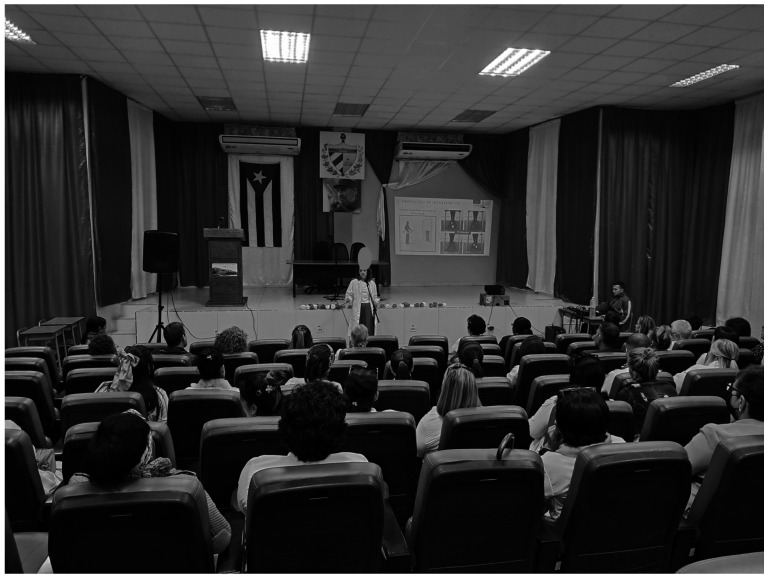
In-person training course in the Provincial Hospital “Leon Cuervo Rubio”.

As noted above, two members of the UV traveled to Pinar del Río to train local physiotherapists in the intervention protocol. Specifically, four in-person training courses were conducted during the researchers’ stay at the following sites: “5 de Septiembre” Polyclinic (Consolación del Sur), “Primero de Enero” Polyclinic (Consolación del Sur), Pediatric Hospital (Pinar del Río) and Provincial Hospital “Leon Cuervo Rubio” (Pinar del Río) (Figure 1). Workshops and module summaries were provided to health professionals and to key rehabilitative and medical stakeholders from Pinar del Río following the initial virtual sessions. Knowledge was subsequently disseminated to broader local teams, with ongoing online follow-up support. Particular emphasis was placed on the mirror neuron system, including its physiology, the current evidence base, and the implementation of the transferred rehabilitation protocol.

Satisfaction with the training was assessed at the end of the on-site training period using an ad hoc Likert-type questionnaire specifically developed for this international cooperation project. The questionnaire was structured into three dimensions: (i) pedagogical value and instructional design quality (13 items, rated on a 5-point Likert scale ranging from 1 = very low agreement to 5 = very high agreement), (ii) perceived usefulness and satisfaction (9 items rated on the same 5-point Likert scale), and (iii) overall satisfaction (2 items rated on a 0 to 10 numerical scale, with 10 being the maximum score). For each dimension, the score was calculated as the mean of the corresponding item scores. No item is required to reverse coding. The complete questionnaire and its English translation are provided as Supplementary Material File S1.

### 2.5. Recruitment of Participants and Local Professionals

Local partners, including practitioners and physiotherapists, were responsible for identifying and recruiting eligible participants and their family members in accordance with the predefined inclusion criteria. Eligible participants were older adults classified as frail or prefrail according to the Fried criteria, with or without neurological conditions such as stroke, Parkinson’s disease, or traumatic brain injury. Frailty status was assessed using the Fried criteria [18], classifying participants as non-frail, pre-frail, or frail based on five components: unintentional weight loss, exhaustion, low physical activity, slow gait speed, and weak grip strength. Exclusion criteria comprised severe cognitive impairment limiting comprehension of instructions, medical instability, visual impairment, or contraindications to standing or physical activity.

In addition, the Municipal Health and Academic Sector actively promoted participation in both online and on-site training sessions led by the UV. Healthcare professionals and faculty members from the Degree of Rehabilitation were involved in the training program. All participants provided written informed consent to take part in the initiative.

### 2.6. Clinical Trial Implementation

The main objective of this study was to assess the feasibility of transferring the proposed intervention (i.e., Virtual Walking and therapeutic exercise). Two physiotherapists from the UV traveled to Pinar del Río to train and supervise the local professionals during a one-month on-site period, ensuring standardized implementation of the intervention protocol across centres. This on-site supervision aimed to guarantee procedural consistency among local physiotherapists. The training program included practical instruction in both assessment and intervention procedures. Local healthcare professionals received theoretical and hands-on training in system setup, participant positioning, safety considerations, and progression criteria, supported by standardized written materials and visual aids.

**Figure 2:**
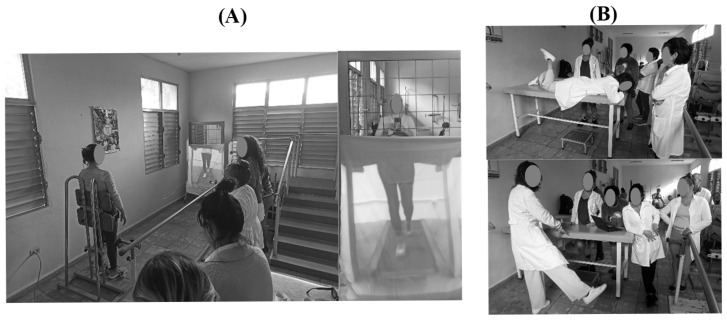
Experimental procedure: (**A**) Virtual reality implementation; (**B**) Exercise intervention.

The rehabilitation program consisted of a 10 min Virtual Walking session follow individualizedtherapeutic exercises targeting lower-limb strength and coordination (Figure 2). Sessions were conducted three times per week for eight weeks. Local professionals were responsible for daily operations, patient assessment, and ongoing data collection. All rehabilitative and digital materials were provided by the UV team. All activities were conducted with respect for participants’ dignity and sociocultural diversity, and in accordance with applicable ethical guidelines.

#### 2.6.1. Virtual Walking Setup

Virtual Walking was delivered using a low-cost, non-immersive visual system composed of a conventional mirror reflecting the participant’s upper body, a height-adjustable external screen positioned below waist level, and a standard video projector connected to a laptop computer. The visual stimulus consisted of pre-recorded videos depicting lower-limb walking movements, previously captured by healthy individuals with varying anthropometric characteristics. During the first session, the projected lower limbs were visually scaled and spatially aligned to each participant’s body dimensions, facilitating integration of the projected legs with the mirrored upper body and eliciting a bodily illusion of a complete, moving body. Participants remained standing and were instructed to observe and perceptually incorporate the projected legs into their own body representation. The system was characterized as low-cost due to its reliance on commercially available equipment (mirror, projector, screen, and laptop) and the absence of proprietary software or immersive virtual reality hardware, thereby facilitating replication in resource-limited settings.

#### 2.6.2. Therapeutic Exercise

Following the Virtual Walking session, participants performed an individualized therapeutic exercise program lasting approximately 30 min, targeting gait, coordination, balance, muscle strength, and lower-limb flexibility. Aerobic exercise was not explicitly prescribed, as the primary visual illusion focused on gait; it was therefore assumed that aerobic stimulation was partially achieved through this modality, complemented by the other exercise components in the therapeutic protocol. Although the Virtual Walking component was performed in a standing position using a support stand that did not require harnesses or external support, therapeutic exercises were individually adapted according to each participant’s functional status, fatigue levels, and balance capacity. When necessary, exercises were performed in a seated position, rest periods were extended, and progression of load and task complexity was adjusted daily, ensuring a moderate perceived exertion level on the Borg scale [19].

### 2.7. Monitoring and Outcome Measures

Monitoring included weekly online meetings during the initial implementation phase and daily supervision during the one-month on-site period. Adherence was tracked using attendance logs and signed registration records. In addition, bi-monthly online meetings with the local partners were conducted post-implementation to support ongoing program adjustment and the timely resolution of incidents.

As part of the local implementation, healthcare professionals were trained to administer validated clinical outcome measures incorporated into the rehabilitation protocol. These included the 10-Meter Walking Test (10MWT) to assess gait speed [20]; the 6-Minute Walk Test (6MWT) to assess aerobic capacity [21]; the Timed Up and Go test (TUGT) to assess functional mobility [22]; the Fried Frailty Criteria to assess frailty status in older adults [18]; and the Visual Analog Scale (VAS) to assess pain intensity [23]. The purpose of these assessments was to examine the feasibility of applying the protocol across different populations in this region and to ensure that the local team could integrate both assessments and interventions into their routine clinical practice. Local assessors were licensed physiotherapists, and detailed written instructions were provided. Beyond the initial one-month training period, continuous communication was maintained via online channels to ensure standardization throughout the assessment process. Collectively, these procedures contributed to the reliability of locally conducted assessments.

### 2.8. Data Analysis

Statistical analyses were conducted using SPSS version 28.0 (IBM Corp., Armonk, NY, USA). Given the small sample size (n = 11) and the non-normal distribution of the outcome variables, nonparametric statistical methods were applied. Pre-post comparisons were performed using the Wilcoxon signed-rank test for paired samples. Quantitative data were reported as medians and interquartile ranges (IQRs). Change scores were calculated as individual paired differences (post–pre). The level of statistical significance was set a priori at *p* < 0.05 (exact two-tailed). Effect sizes of the Wilcoxon signed-rank test were calculated using the r statistic (r = Z/$\sqrt{N}$) and interpreted as small (r = 0.1), moderate (r = 0.3), or large (r = 0.5) effects, where applicable. In addition, graphical representations were generated to illustrate individual trajectories on each assessment scale, as these plots were considered complementary to the inferential analysis and particularly informative given the sample’s heterogeneity and the exploratory nature of the study.

Categorical variables were analyzed descriptively, due to the paired pre–post design and the small sample size.

## 3. Results

The program was successfully implemented, facilitated by close collaboration with local partners and expert communities, and utilized available technologies through an open-science framework. Two polyclinics were fully equipped and now provide rehabilitation services, including the experimental protocol, with local personnel trained to address technical challenges and ensure continuity of care.

### 3.1. Training Satisfaction Results

Eighty-five health professionals (sixty-nine women and sixteen men; mean (SD) age = 40.03 (10.68) years) participated in the training activities and completed a satisfaction survey. The healthcare professionals reported that the training had excellent pedagogical value and instructional design quality (4.94 (0.17) out of 5). They also rated the training as highly useful (4.95 (0.13) out of 5). Finally, overall satisfaction with the training and the instructors was extremely high (10 out of 10).

### 3.2. Intervention Results

Baseline and post-intervention functional assessments of participants enrolled in the Virtual Walking program were conducted by local healthcare professionals. A total of two centers participated in the implementation. The preliminary results presented here involve 11 participants (8 men and 3 women), with a mean age of 77.09 (10.00) years. The mean body weight was 66.42 (10.50) kg, mean height 1.71 (0.10) m, and mean body mass index 22.94 (4.03). The diagnostic distribution included eight stroke survivors, one traumatic brain injury, and two individuals with Parkinson’s disease. All the participants completed all 24 intervention sessions.

#### 3.2.1. Functional Assessments

As shown in Table 2, walking endurance improved significantly, whereas no statistically significant changes were observed in gait speed or functional mobility. Wilcoxon signed-rank tests were performed to examine pre-post changes in 10MWT, 6MWT, and TUGT.

Specifically, endurance, as assessed by the 6MWT, improved significantly following the intervention (Z = −2.312; *p* = 0.019; r = 0.70). Gait speed, as measured by the 10MWT, showed no significant improvement (Z = −1.869; *p* = 0.06). Similarly, TUG scores (i.e., functional mobility) did not change significantly from baseline to post-intervention (Z = −1.156; *p* = 0.28).

**Figure 3:**
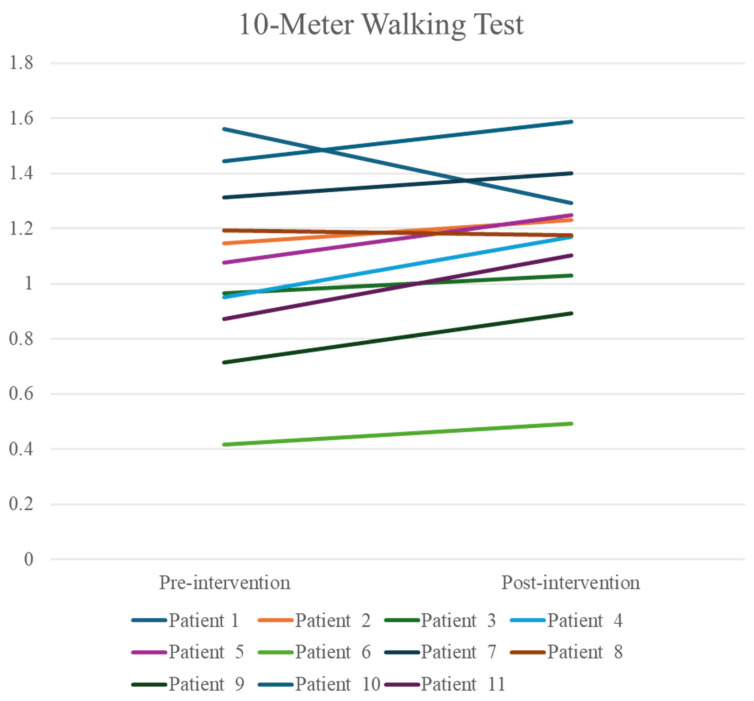
Individual responses in 10-Meter Walking Test. Units are presented in m/s.

**Figure 4:**
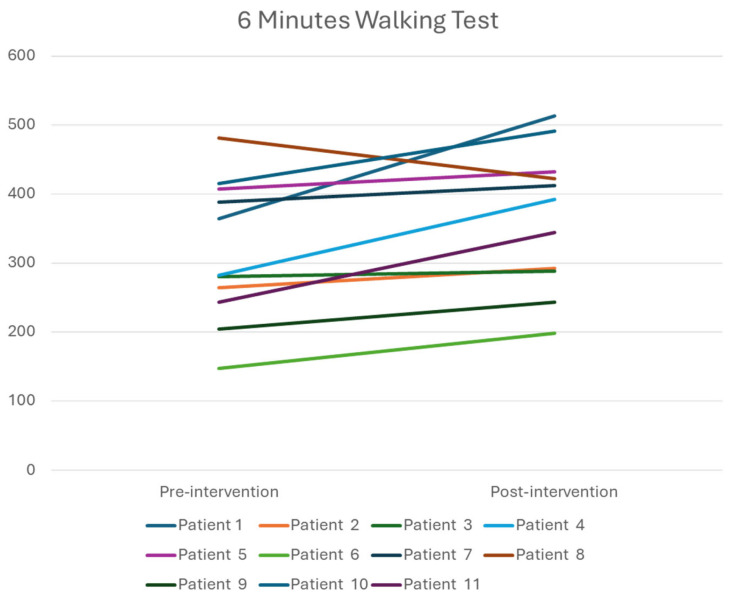
Individual responses in the 6-Minute Walk Test. Units are presented in m.

**Figure 5:**
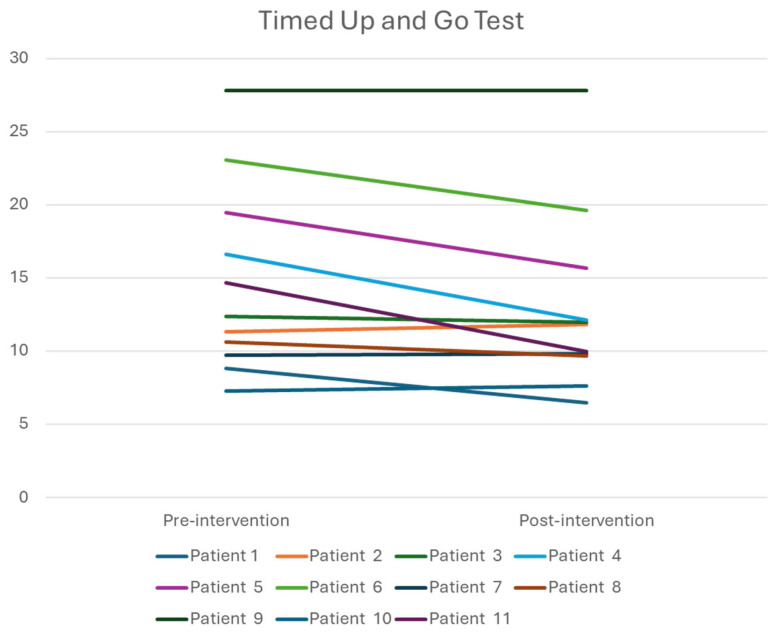
Individual responses in the Timed Up and Go test. Units are presented in s.

Given the clinical heterogeneity of the sample, individual response profiles are presented in Figure 3, Figure 4 and Figure 5 to illustrate variability in outcomes. Specifically, patients 8 and 11 had Parkinson’s disease, patient 7 had a traumatic brain injury, and the remaining participants were stroke survivors. Visual inspection of individual trajectories did not reveal a consistent pattern of responses across neurological diagnoses.

#### 3.2.2. Use of Walking Aids

At pre-intervention, eight participants completed the 10MWT unassisted, while three participants required a cane. By post-intervention, 10 participants walked independently, and only 1 used a cane. Similar results were observed in the TUGT, including reduced reliance on walking aids for standing, walking, and sitting. For the 6MWT, ten participants completed the test without support at both pre- and post-intervention, while one participant required support at both time points.

#### 3.2.3. Pain

Pain outcomes remained stable: seven participants reported no pain, while four reported persistent pain at both pre- and post-intervention. No differences between time-points were observed.

#### 3.2.4. Frailty Status

At pre-intervention, four participants were classified as non-frail (robust), four as pre-frail, and three as frail. At post-intervention, most participants were classified as pre-frail (n = 10), and one as frail. When analyzing individual trajectories, two participants improved from frail to pre-frail; one frail participant and one pre-frail participant remained stable; and all initially robust participants shifted to pre-frail.

## 4. Discussion

This project was designed in response to the lack of resources in Cuba, with the specific aim of providing training and implementing recently evidenced intervention strategies, thereby improving the quality of rehabilitation care.

### 4.1. Clinical Intervention Protocol

The implementation of a tailored, low-cost rehabilitation protocol combining mirror neuron activation through bodily illusion and task-specific therapeutic exercise was feasible in this setting and was associated with improvements in functional abilities and engagement in the rehabilitation process. Individualisation was operationalised by adjusting task difficulty, exercise intensity progression, and walking speed demands based on participants’ functional capacity, observed performance, fatigue, and safety considerations. Progression decisions were made session by session to ensure feasibility while maintaining therapeutic challenge. From an implementation perspective, the protocol prioritized adaptability over strict standarisation, allowing clinicians to modulate intensity and task demands while preserving core therapeutic principles.

In this sense, conventional gait training remains a cornerstone of rehabilitation and is essential whenever safely feasible. However, in older adults with neurological disorders, frailty, balance impairments, or reduced exercise tolerance, repetitive real-world gait practice may be limited by fatigue or fall risk. In this context, bodily illusion therapies provide a valuable complementary approach, activating motor and sensorimotor mechanisms without requiring high physical demand [24]. Visual bodily illusion may amplify motor learning processes and optimize motor control, potentially enhancing the functional impact of physical training without inducing excessive fatigue.

The intervention also enhanced healthcare providers’ knowledge of traditional and recently evidenced strategies and promoted active participation among users. Healthcare professionals received both material resources and theoretical-practical training, increasing their capacity to plan and deliver evidence-based rehabilitation interventions [17]. Beyond immediate patient care, the training was associated with very high satisfaction scores, reflecting clarity, usability, and confidence in applying the protocol. From an implementation perspective, high satisfaction is particularly relevant as it is associated with improved understanding and adherence to protocols in real-world settings. The program also strengthened teaching practices among local health educators, empowering family members and caregivers to participate directly in patient recovery and alleviating physical and emotional burdens. Additionally, the local health system benefited from two fully equipped polyclinics with reusable rehabilitation materials, which may reduce costs associated with dependency and hospital admissions.

### 4.2. Preliminary Outcomes and Limitations

Within this context, the preliminary results of this study suggest that no statistically significant changes were observed in gait speed or balance-related performance, and pain outcomes remained stable. Regarding frailty status, although the number of participants classified as “frail” decreased, there was a general shift towards “pre-frailty” among initially robust. This pattern suggests heterogeneity in response to the intervention, with some individuals showing no improvement or even a slight decline in specific components (e.g., gait speed, grip strength, or fatigue). Consequently, these findings highlight that the intervention may not uniformly impact frailty status across all participants.

To contextualize the clinical relevance of these functional changes, previously reported minimal clinically important differences (MCID) values were considered. For gait speed, as assessed by the 10MWT, MCID thresholds of approximately 0.10 m/s have been reported in older adults and stroke survivors [25,26], consistent with the mean change observed in this study. For walking endurance measured by the 6MWT, typical MCID values range from 20 to 30 m in older adults and from 35 to 40 m in post-stroke populations [25,27]. Interestingly, the median improvement observed in this study was 109.5 m, thus exceeding these thresholds.

As this was a preliminary, non-controlled implementation study, no conclusions can be drawn regarding whether these gains were predominantly driven by cardiorespiratory adaptations, neuromotor improvements, or their relative contribution. The small sample size and use of nonparametric pre-post analyses limit generalizability and preclude causal inference. Given the exploratory nature of the study and the small sample size, these results should be interpreted as indicative trends rather than definitive evidence of effectiveness.

### 4.3. Implications for Future Rehabilitation Policy and Healthcare in Cuba

The positive effects observed align with previous research highlighting the value of international cooperation in healthcare capacity building and technology transfer [17]. Our findings suggest that function-oriented interventions grounded in mirror neuron activation and task-specific therapeutic exercise can be feasibly implemented in real-world, resource-constrained settings. Importantly, this study demonstrates real-world applicability in a low-resource environment, although the observed functional improvements should be considered preliminary and context-specific.

The use of digital tools and open-science approaches may facilitate broader dissemination and adaptability [28], despite logistical barriers in Cuba relative to other settings [29,30]. In this study, digital resources primarily supported training and supervision, and their broader impact on scalability and sustainability requires confirmation in future research.

The program’s participatory structure, involving users, local clinicians, and educators, emphasizes the importance of empowerment and local ownership for sustainability [31]. While high satisfaction levels and active engagement were observed, the long-term sustainability of these effects and their impact on routine clinical practice were not formally assessed and warrant further investigation. A major strength of the initiative is its replicable framework, with clearly defined session duration, exercise type, and therapist-led individualized adaptation criteria, and provision of all methods and materials required for replication, including local infrastructural, organizational, and human resource conditions.

Regarding sustainability, the integrative methodology and knowledge transfer were effective not only clinically but also in promoting social inclusion, equity, and alignment with the Sustainable Development Goals (SDGs 3, 4, and 10) [32]. Although conceptual alignment was achieved, the direct contribution to long-term social or system-level outcomes was not quantitatively assessed. The integration of digital technologies allows continuous remote support and local leadership, representing a potential mechanism for sustaining benefits rather than a confirmed outcome.

### 4.4. Strengths, Limitations and Future Studies

Several limitations must be acknowledged. Poor digital infrastructure in Pinar del Río limited independent access to training materials, necessitating the dissemination of USBs and group sessions using projectors. Limited electricity further complicated data processing [33]. Transportation challenges, particularly for participants from rural areas, affected attendance and reflected structural barriers inherent to low-resource rehabilitation settings. Delays in equipment delivery necessitated adaptation using locally available materials, demonstrating that high-cost technology is not essential to achieving intervention goals.

Additionally, key functional outcomes, such as gait speed and balance, did not show statistically significant improvements, which may be due to the small sample size, clinical heterogeneity, and limited statistical power. The small sample size and use of nonparametric pre-post analyses limit generalizability and preclude causal inference. Future studies should examine long-term outcomes and mechanisms sustaining innovations in local health systems.

This project provides a replicable model for international cooperation in rehabilitation, establishing a foundation for the systematic application of bodily illusion therapies and digital-based rehabilitation strategies in resource-limited communities. Observed trends toward functional improvements, particularly in walking endurance, support the potential clinical value of such interventions and highlight the need for larger, controlled studies. Taken together, these limitations define important directions for future research, rather than definitive evidence of clinical effectiveness. Future works should include expanded sample sizes, longitudinal follow-up to assess the durability of functional gains, low-cost technology innovations, and efforts to overcome infrastructural barriers and foster participation among professionals and families. Moreover, future studies could explore applicability in other age or pathological groups, including children. The barriers identified underscore the potential value of complementary rehabilitation strategies that require minimal equipment and are feasible for home implementation. Home-based or caregiver-assisted exercise programs that incorporate task-oriented training and action observation principles may enhance accessibility and continuity of care. Future studies should explore hybrid models combining clinic-based and home-based interventions to improve adherence and scalability.

## 5. Conclusions

Without downplaying the above-mentioned limitations, this cooperation project between the UV (Spain) and the healthcare and academic system of Pinar del Río (Cuba) demonstrates the feasibility of implementing innovative, low-cost rehabilitation strategies in resource-limited settings. Through a combination of digital and face-to-face training, tailored therapeutic protocols, and collaborative local engagement, the program was associated with short-term, preliminary improvements in endurance, with no statistically significant improvements in the remaining studied functional outcomes. Furthermore, the project promotes initial capacity building among healthcare providers and increased involvement of family members in the rehabilitation process.

The present findings should not be interpreted as evidence of long-term effectiveness, sustainability, or system-level impact. Rather, they reflect the successful initial transfer and short-term implementation of an evidence-informed intervention in two local clinical sites. No quantitative indicators of health system performance were collected, and the limited sample size and scale of implementation preclude broader conclusions regarding health system strengthening.

Nevertheless, this initiative illustrates how scientific advances and evidence-informed rehabilitation practices can be contextually adapted, locally implemented, and initially maintained through structured training and supervision in low-resource environments. These preliminary findings provide a foundation for future large controlled studies, longitudinal follow-up evaluations, and formal assessment of scalability and sustainability.

## Figures and Tables

**Table 1:** Topics addressed in the meetings.

Pre-Stay Meetings	
Meeting 1	Defining Project Objectives Clarified the overarching goals of the cooperation initiative between Spain and Cuba. Ensured alignment with institutional priorities in both countries. Agreed and focusing on rehabilitation protocols combining Virtual Walking and therapeutic exercise.
Meeting 2	Action Plan Development Drafted a phased implementation plan. Identified short-term and long-term deliverables. Assigned preliminary responsibilities across partner institutions.
Meeting 3	Identification of Local Needs Cuban representatives presented the main health and rehabilitation challenges in their centers. Discussed populations with a higher prevalence of frailty and neurological disorders. Prioritized areas where external support and training were most required.
Meeting 4	Assessment of Available Resources Reviewed clinical infrastructure and technological capacity at participating centers. Identified gaps in equipment, space, and digital access. Determined minimum requirements for intervention feasibility.
Meeting 5	Logistical Arrangements for Training and Roll-Out Outlined travel, accommodation, and visa processes for the visiting team. Defined local hosting arrangements. Scheduled initial training workshops and practical demonstrations.
Meeting 6	Practical Issues Related to Residency and Field Activities Addressed housing and transport arrangements during the one-month stay. Agreed on protocols for local supervision and coordination. Discussed health and safety considerations.
Meeting 7	Educational Materials Selection and Adaptation Reviewed draft versions of video-based modules. Adapted content to local cultural and linguistic contexts. Finalized the nine thematic modules to be included.
Meeting 8	Strategies to Overcome Infrastructure Limitations Brainstormed solutions for intermittent internet access. Considered offline alternatives to guarantee continuity of training (offline versions of materials). Agreed on the provision of basic technical equipment by the project.
**Post-residence meetings**	
Meeting 9	Reception of Technical Equipment and Installation Confirmed arrival of projectors, therapeutic exercise equipment, and digital devices. Reported minor difficulties during equipment setup. Provided remote guidance to local technicians to ensure proper installation.
Meeting 10	Troubleshooting Baseline Assessment Discussed initial challenges encountered by local teams during patient assessment. Clarified scoring protocols and data entry procedures. Reached consensus on standardized methods to ensure consistency across centers.
Meeting 11	Data Collection and Program Evaluation Reviewed strategies for data management and secure transfer. Emphasized the descriptive nature of the analyses, given sample heterogeneity. Planned periodic updates to monitor ongoing implementation outcomes.

**Table 2:** Group results for functional assessment.

Test	Pre-Intervention, Median (IQR)	Post-Intervention, Median (IQR)	Median Paired Change (Post-Pre)	*p* Value	Effect Size (r)
10-Meter Walking Test (m/s)	1.08 (0.44)	1.18 (0.26)	0.10	0.06	-
6-Minute Walk Test (m)	282.60 (164.60)	392.10 (144.00)	109.5	0.019	0.70
Timed Up and Go test (s)	11.31 (6.90)	11.81 (6.01)	0.5	0.28	-

IQR: Interquartile range. N = 11 paired observations (no ties); exact two-tailed *p*-values reported; effect size r calculated as r = Z/$\sqrt{N}$.

## Data Availability

The data supporting the findings of this study are available from the corresponding author upon reasonable request, as they are not publicly available due to privacy and ethical restrictions.

## Supplementary Materials

The following supporting information can be downloaded at: https://www.mdpi.com/article/10.3390/healthcare14030352/s1, File S1: Cooperation project satisfaction survey (Spanish version and English translation).

## Abbreviations

The following abbreviations are used in this manuscript:

6MWT	6-Minute Walk Test
10MWT	10-Meter Walking Test
TUGT	Timed Up & Go test
SD	Standard Deviation
SDGs	United Nations Sustainable Development Goals
UV	University of Valencia
VAS	Visual Analog Scale
