# Supplementary material for: Implementation of an Intervention Program Based on Virtual Walking and Therapeutic Exercise in Cuba: A Feasibility Study
Source: Healthcare (Basel). 2026 Jan 30;14(3):352. doi: 10.3390/healthcare14030352 (PMC12896961; doi:10.3390/healthcare14030352)
Supplement: Supplementary file 1 [file healthcare-14-00352-s001.zip › healthcare-4093209-supplementary.pdf]

ENCUESTA DE SATISFACCIÓN PROYECTO COOPERACIÓN
[SPANISH VERSION]

Nos interesa conocer tu opinión sobre el proyecto de cooperación "TRANSFERENCIA DE UN PROTOCOLO DE MARCHA VIRTUAL Y EJERCICIO TERAPÉUTICO PARA PERSONAS CON ALTERACIÓN DE LA CAPACIDAD FUNCIONAL EN PINAR DEL RÍO" llevado a cabo en el marco de las ayudas (VII Convocatoria) a Proyectos de Cooperación al Desarrollo de la *Universitat de València*, para realizar un análisis de la satisfacción de los terapeutas con la formación contemplada en dicho proyecto.
Te agradecemos de antemano tu valiosa colaboración.

PARTE 1- DATOS GENERALES

1. **Sexo:**

   ▪ Varón ▪ Mujer

2. **Edad:** ……. Años
3. **Estudios (indique el título de máximo nivel):**
4. **Categoría profesional:**

PARTE 2- VALOR PEDAGÓGICO Y CALIDAD DEL DISEÑO

En este cuestionario se pregunta tu opinión sobre el valor pedagógico y la calidad de la formación. Por favor, señala tu grado de acuerdo con las siguientes afirmaciones:

	1<br>Muy en desacuerdo	2<br>Bastante desacuerdo	3<br>Ni acuerdo ni desacuerdo	4<br>Bastante acuerdo	5<br>Muy de acuerdo
1. Adecuación de los temas en los que se divide la formación					
2. Volumen de información					
3. Estructura					
4. Fragmentación del contenido					
5. Claridad de la información					
6. Calidad de las docentes					
7. La formación me ha ayudado a profundizar en mis conocimientos sobre el tema					
8. El contenido de la formación se corresponde con lo esperado					
9. La formación recibida me ayuda a entender mejor la intervención del proyecto					
10. La formación recibida estimula mi interés por la intervención del proyecto					
12. La formación recibida ha promovido el desarrollo de mis competencias técnicas (ej. Estrategias de intervención) como no técnicas (p.e., comunicación y cuidado de pacientes)					
13. La formación recibida me ha facilitado disponer de más oportunidades de práctica y de aprendizaje de nuevas técnicas					
14. La formación recibida ha constituido un recurso educativo que ha promovido un aprendizaje de calidad en la materia.					
15. La formación recibida me ha permitido adquirir nuevas competencias en el abordaje de los pacientes con alteración funcional					

PARTE 3- UTILIDAD Y SATISFACCIÓN

En este cuestionario se pregunta sobre la utilidad y satisfacción con la formación recibida Por favor, señala tu nivel de acuerdo con cada una de las siguientes afirmaciones (1 = totalmente en desacuerdo y 7=totalmente de acuerdo). Trata de responder a todos los enunciados.

	1<br>Muy en desacuerdo	2<br>Bastante desacuerdo	3<br>Ni acuerdo ni desacuerdo	4<br>Bastante acuerdo	5<br>Muy de acuerdo
1. Me ha resultado útil					
2. Me ha ayudado a profundizar mis conocimientos sobre el tema					
3. Me ha dado más control (autonomía)					
7. Cumple con mis necesidades					
8. Cumple con lo que esperaba de ella					
9. Estoy satisfecho/a con formación recibida					
25. Se lo recomendaría a mis compañeros/as					
29. Creo que es necesario tener este tipo de formaciones de terapeutas de otros países					
30. Repetiría más formaciones de las mismas docentes					

PARTE 4- SATISFACCIÓN GLOBAL

En su conjunto, la formación recibida merece una valoración global de:	1	2	3	4	5	6	7	8	9	10
En su conjunto, las docentes merecen una valoración global de:	1	2	3	4	5	6	7	8	9	10

COOPERATION PROJECT SATISFACTION SURVEY
[ENGLISH TRANSLATION]

We are interested in knowing your opinion about the cooperation project "TRANSFER OF A VIRTUAL GAIT PROTOCOL AND THERAPEUTIC EXERCISE FOR PEOPLE WITH ALTERATION OF FUNCTIONAL CAPACITY IN PINAR DEL RÍO", which has been funded by the 7th Grants for Cooperation Projects aimed at Development of the *University of Valencia*, to analyse the satisfaction of the therapists with the training included in this project.
We thank you in advance for your valuable cooperation.

PART 1- GENERAL DATA

1. **Sex:**
   - Male
   - Women
2. **Age:** ……. Years
3. **Education (indicate the highest-level degree):**
4. **Professional category:**

PART 2- PEDAGOGICAL VALUE AND DESIGN QUALITY

This questionnaire asks for your opinion on the pedagogical value and quality of the training. Please indicate your degree of agreement with the following statements:

	1 Strongly disagree	2 Quite a bit of disagreement	3 Neither agreement nor disagreement	4 Quite a bit of agreement	5 Strongly agree
1. Adequacy of the topics into which the training is divided					
2. Volume of information					
3. Structure					
4. Content fragmentation					
5. Clarity of information					
6. Quality of teachers					
7. The training has helped me to deepen my knowledge on the subject					
8. The content of the training corresponds to what is expected					
9. The training I received helps me to better understand the project intervention					
10. The training I received stimulates my interest in the intervention of the project					
12. The training received has promoted the development of my technical skills (e.g. intervention strategies) as well as non-technical skills (e.g. communication and patient care)					
13. The training I received has given me more opportunities to practice and learn new techniques					
14. The training received has been an educational resource that has promoted quality learning in the subject.					
15. The training I received has allowed me to acquire new skills in the approach to patients with functional alteration					

PART 3- USEFULNESS AND SATISFACTION

This questionnaire asks about the usefulness and satisfaction with the training received. Please indicate your level of agreement with each of the following statements (1 = strongly disagree and 7 = strongly agree). Try to respond to all statements.

	1 Strongly disagree	2 Quite a bit of disagreement	3 Neither agreement nor disagreement	4 Quite a bit of agreement	5 Strongly agree
16. I found the training helpful					
17. It has helped me to deepen my knowledge on the subject					
18. It has given me more control (autonomy)					
19. It meets my needs					
20. It fulfills what I expected of her					
21. I am satisfied with the training I received					
22. I would recommend it to my colleagues					
23. I think it is necessary to have this type of training for therapists from other countries					
24. I would repeat more training sessions given by the same teachers					

PART 4- OVERALL SATISFACTION

The training received deserves an overall assessment of: 1 2 3 4 5 6 7 8 9 10

The teachers deserve an overall assessment of: 1 2 3 4 5 6 7 8 9 10
